# A Comprehensive Review of the Application and Potential of Straw Biochar in the Remediation of Heavy Metal-Contaminated Soil

**DOI:** 10.3390/toxics13020069

**Published:** 2025-01-21

**Authors:** Lei Xu, Feifei Zhao, Jianbiao Peng, Mingfei Ji, B. Larry Li

**Affiliations:** 1 Henan Province Engineering Research Center of Environmental Laser Remote Sensing Technology and Application, Nanyang Normal University, Nanyang 473061, China; terry__xp@163.com; 2 College of Water Resources and Modern Agriculture, Nanyang Normal University, Nanyang 473061, China; pjb126com@163.com (J.P.); jimfdy@gmail.com (M.J.); bai-lian.li@ucr.edu (B.L.L.); 3 Ecological Complexity and Modeling Laboratory, Department of Botany and Plant Sciences, University of California–Riverside, Riverside, CA 92521, USA

**Keywords:** biochar, heavy metals, soil remediation, application potential

## Abstract

With the rapid development of industry and agriculture, soil heavy metal contamination has become an important environmental issue faced today and has gradually attracted widespread attention. Finding a cheap, widely available, and biodegradable material that can promote crop growth and stabilize heavy metals has become a research focus. Crop straw biochar, due to its high specific surface area, rich surface functional groups, and high cation exchange capacity (CEC), has shown good effects on the remediation of inorganic and organic pollutants in the environment. This article reviews recent research on the use of crop straw biochar for soil heavy metal contamination remediation, providing a detailed analysis from the preparation, characteristics, modification of crop straw biochar, mechanisms for reducing the toxicity of heavy metals in soil, and its application and risks in remediating heavy metal-contaminated soils. It also comprehensively discusses the potential application of crop straw biochar in the remediation of heavy metal-contaminated soils. The results show that crop straw biochar can be used as a new type of immobilizing material for the remediation of heavy metal-contaminated soils, but there are issues with the remediation technology that needs to be optimized and innovated, which poses challenges to the widespread application of crop straw biochar. In the future, efforts should be strengthened to optimize and innovate the application technology of crop straw biochar, conduct research on the remediation effects of cheap modified crop straw biochar and the co-application of crop straw biochar with other immobilizing materials on heavy metal-contaminated soils, and carry out long-term monitoring of the effects of crop straw biochar in soil heavy metal remediation in order to achieve the goal of ensuring food safety and the rational use of solid waste.

## 1. Introduction

As a vital component of terrestrial ecosystems, soil is an irreplaceable environmental factor for human and animal habitation and serves as the fundamental guarantee for food security and human health. It plays a crucial role in protecting the environment and maintaining ecological balance. In recent years, soil contamination has become increasingly severe due to a series of anthropogenic activities such as industry and agricultural production [1]. This has led to a decline in the quality of necessities like grain and vegetables, directly endangering human health. With the rapid development of the social economy, China faces a serious issue of heavy metal contamination in its soil environment, including excessive levels of chromium (Cr), cadmium (Cd), lead (Pb), mercury (Hg), arsenic (As), copper (Cu), zinc (Zn), and nickel (Ni) [2]. The situation of soil heavy metal contamination in China is not optimistic. According to the “National Soil Pollution Bulletin” released by the Ministry of Environmental Protection and the Ministry of Land and Resources in 2014, 19.4% of arable land in China has been polluted to varying degrees [3].

With the improvement of people’s living standards, ecology and environmental protection are increasingly valued. Western countries have invested plenty of manpower and resources in the remediation of polluted soil, with the remediation of heavy metal-contaminated soil as an important part of this effort [4,5]. It is preliminarily predicted that the compound annual growth rate of the revenue of China’s environmental protection industry will reach 10% from 2024 to 2029, and the revenue scale of China’s environmental protection industry is expected to exceed CNY 4 trillion (about 580 billion US dollars, calculated at the current exchange rate) by the end of 2029 according to the environmental industry development goals proposed in the “Action Outline for Accelerating the High-Quality Development of the Ecological Environmental Protection Industry and Deeply Fighting Pollution (2021–2030)” [6,7]. Therefore, whether from the perspective of environmental protection needs or from a commercial standpoint, the remediation of heavy metal-contaminated soil has an extremely broad application prospect.

In the remediation of heavy metal-contaminated soil, common remediation methods include the following: engineering measures (soil replacement, deep tillage), physical-chemical remediation (electrokinetic remediation, electrothermal remediation), chemical remediation (soil washing, soil immobilization), and biological remediation (phytoremediation, microbial remediation) [8,9,10,11,12]. Immobilization refers to the process of changing the distribution of heavy metals in the soil, reducing their mobility and bioavailability, and thus reducing their ecological risk. Compared with other remediation methods, it has the advantages of lower remediation costs, simple operation, and a shorter remediation cycle, making it more suitable for the remediation of large areas of polluted soil [5,13,14,15]. Common immobilizing materials include alkaline materials (fly ash, calcium carbonate, etc.), organic matter (organic compost, urban sludge, etc.), metals or metal oxides (zero-valent iron, ferrous sulfate, etc.), and clay minerals [16,17,18,19,20]. However, these immobilizing agents have drawbacks such as gradually decreasing remediation effects over time, containing a certain amount of pollutants themselves or high costs. Therefore, finding an immobilizing agent that is inexpensive, widely available, biodegradable, and capable of promoting crop growth and stabilizing heavy metals has become a research focus.

Biochar refers to a class of porous, high-specific surface area, highly aromatic, and relatively stable physical–chemical properties of carbon materials formed by the pyrolysis of biomass or biodegradable waste, such as agricultural waste, trees, plant tissues, or animal manure, under oxygen-limited or oxygen-free conditions at high temperatures (400 °C to 700 °C) [21,22]. These unique physicochemical properties of biochar have led to its widespread application in many fields such as energy recovery, carbon fixation, soil remediation, and wastewater treatment [23]. Additionally, the simple production process and low raw material costs have led to an increasing number of studies on the preparation, modification, and application of biochar, with a trend of rising year by year. The type of preparation material determines the properties, functions, and application range of biochar. Under the same preparation conditions, biochar derived from different types of biomass pyrolysis may exhibit completely different physicochemical properties and functional characteristics [24,25]. Studies have shown that straw, as an agricultural waste, contains fewer harmful wastes (heavy metals, organic pollutants, etc.) compared to municipal waste, industrial waste, and sludge [26]. The environmental risk of applying biochar made from straw in the field is relatively low, hence the application of straw biochar in the remediation of heavy metal-contaminated soil is more frequent [27]. More importantly, compared to other biomasses (such as forestry residues and municipal solid waste), straw has the characteristics of being abundant and widely sourced. Thus, straw biochar holds a bright future and great potential for the remediation of soils contaminated with heavy metals.

This study primarily focuses on international research regarding the preparation process, characteristics, modifications, mechanisms of adsorbing heavy metal ions, influencing factors, and applications of straw biochar in the remediation of heavy metal-contaminated soil. This research offers a theoretical basis for elucidating the role of biochar in the remediation of heavy metal-contaminated soils and for uncovering the mechanisms by which biochar contributes to the remediation of such soils. Additionally, it can offer scientific evidence for the sustainable use of modified straw biochar in soil.

## 2. Preparation and Basic Characteristics of Straw Biochar

The preparation methods for biochar mainly include conventional pyrolysis, hydrothermal carbonization, and gasification, with conventional pyrolysis more widely applied in the preparation of biochar [28]. Conventional pyrolysis can be divided into two categories based on temperature control: fast pyrolysis and slow pyrolysis [29,30]. Fast pyrolysis (residence time between 0.5 and 5 s) promotes the rapid decomposition of organic matter, resulting in relatively lower biochar yield [29]. Slow pyrolysis (residence time between 600 and 6000 s) involves adding biomass to the reactor after the start of pyrolysis, with a slower temperature rise rate, and obtaining biochar after a longer residence time. Slow pyrolysis not only yields more biochar but also produces biochar with a more abundant carbon content compared to fast pyrolysis [31].

Biochar prepared by slow pyrolysis from straw is mostly alkaline, and the pH value gradually increases with the increase in pyrolysis temperature, which is related to the formation of alkaline oxides and carbonates from alkali metals (K, Na) and alkaline earth metals (Mg, Ca) in the biomass material during the pyrolysis process [32]. There are significant differences in the ash content and elemental composition of biochar from different sources. The ash content of straw biochar ranges from 11.6% to 33.7%, higher than that of wood charcoal and bamboo charcoal (<10%) (Table 1), and the ash content of straw biomass raw materials often determines the pH value of the biochar [33]. Meanwhile, ash is a key component that can affect the functionality of biochar; the ash in biochar can provide essential nutrients for plant growth. Straw biochar generally has a high cation exchange capacity, and its value increases with temperature; therefore, adding straw biochar to the soil can increase the CEC of soil [34]. Compared to biochar prepared from wood, biochar prepared from straw is relatively more dense and more prone to fragmentation [35]. In the remediation of heavy metal-contaminated soils, biochar with smaller particle sizes is more conducive to the immobilization of heavy metals such as cadmium, lead, and zinc [36]. Studies suggest that the ratio of lignin to cellulose content may be one of the factors affecting the particle size distribution of biochar [35]. In addition, straw biochar has a high specific surface area (0.6–355 m^2^/g) and complex pore structure (biochar pore size can generally be divided into three levels: pore size greater than 50 nm, pore size between 2 and 50 nm, and pore size less than 2 nm) [37]. By observing the surface of biochar with scanning electron microscopy (SEM), abundant tubular structures can be found, which originate from the vascular and tracheal structures in the material [38]. In addition, some micropores can be observed on the surface of the material, which are related to the release of pyrolysis gases during the pyrolysis process. Generally, an increase in pyrolysis temperature increases the specific surface area of biochar, but when the temperature is higher than 700 °C, the collapse of pores may lead to a decrease in specific surface area [39,40]. This is primarily because, when the temperature exceeds the optimal level, the pore structure that has already formed in the biochar can be destroyed by the high heat, which is detrimental to the formation of a larger specific surface area and the more abundant pore structure in the biochar [41].

## 3. Modification of Straw Biochar

In the field of soil heavy metal contamination remediation, the pore structure of straw biochar (including pore size distribution and volume), specific surface area, and surface functional groups jointly determine its adsorption efficiency, thereby affecting its adsorption and fixation of polluting components [38]. However, since the biochar prepared from the same type of straw biomass has relatively single nutrients, mainly consisting of organic carbon and phosphorus, and not all of the organic carbon and trace elements in the biochar can be transformed into effective soil fertility, there is a problem of low nutrient content and weak adsorption performance [46,47]. To address the issue of the weak adsorption performance of straw biochar, various modification methods are commonly used in soil heavy metal contamination remediation practices to regulate and control biochar directionally to enhance its adsorption and fixation of pollutants.

Typical biochar modification techniques primarily encompass physical and chemical approaches. Physical modification mainly involves passing steam or other gases (such as nitrogen, oxygen, ammonia, carbon dioxide, etc.) during the pyrolysis of biomass for gasification treatment. Physical modification can improve the hydrophobicity of biochar by promoting the formation of crystalline carbon and optimizing the pore structure, thereby enhancing the performance of biochar [48,49]. Common chemical modification methods consist of acid–base treatment, oxidant treatment, and metal modification (such as the addition of metal salts or metal oxides). Among these, acid–base modification typically enhances the performance of biochar by increasing the number and diversity of functional groups and expanding the specific surface area [50,51]. Oxidant modification hinges on augmenting the variety and amount of oxygen-containing functional groups in biochar to accomplish its modification [52]. Metal modification is capable of enhancing the adsorption capacity of biochar and imparting magnetism to it [53]. The methods commonly used for straw biochar modification are shown in Table 2.

It is evident that physicochemical modification methods primarily alter the physical properties and structural features of biochar. Currently, there are studies using different composite material addition methods for modification, that is, when preparing biochar by the pyrolysis of a certain biomass, another carbon-containing material such as carbon nanotubes, graphene, organic solvents, sludge or wood shavings [60,61,62,63,64]. Among them, carbon nanotubes and organic solvents are expensive and have safety risks in the preparation process, while adding organic waste for biochar modification can achieve waste resource utilization at the same time, making the latter more applicable [65]. Compared with other modification methods, this method belongs to a modification method that integrates the advantages of multiple materials and improves the performance of biochar because the added carbon materials themselves may contain a variety of components. At the same time, the substances contained in the modification materials can be transformed into a part of the biochar as a reaction substrate during the pyrolysis process, making the components of the biochar more abundant [66]. Therefore, the preparation of new-type biochar by the pyrolysis of a biomass as the main material and adding another organic material as a modifier is gradually attracting widespread attention in the academic community.

## 4. The Impact of Straw Biochar on Soil Properties

### 4.1. Soil Physicochemical Properties

Straw biochar has a well-developed pore structure. When added to soil, it can fill the large pores in the soil, dividing them into many smaller pores, increasing the soil’s porosity, thereby improving soil aeration [67]. Compared to straw, straw biochar can improve soil water retention capacity by changing the soil’s porosity and aggregation level [68]. Due to the lower density of straw biochar, increasing its application amount helps to reduce soil bulk density. Zhang et al. conducted a 2-year field experiment showing that, when biochar is added at 20–40 t/hm^2^, it can reduce the soil bulk density by 8.1% to 10.1% in paddy fields [69].

Straw biochar helps to increase the soil CEC, which enhances the soil’s fertility retention ability [70]. Yuan et al. found that rapeseed straw biochar can increase the soil CEC by 15.8% to 25.1% (from 9.12 to 10.56–11.41 cmol/kg) [71]. The extent to which straw biochar enhances the soil CEC depends on the properties of the biochar itself, particularly its preparation temperature. Under conditions of 300–400 °C, the CEC values of straw biochar are higher, and, after 400 °C, as the temperature increases, the CEC tends to decrease [72], the CEC decreases by 50% at 500 °C. Additionally, as straw biochar remains in the soil for a longer time, more oxygen-containing functional groups form on the biochar surface under oxidation, thereby increasing the soil CEC [73].

Straw biochar can effectively raise the pH value of acidic soils and reduce soil acidity [74]. This is mainly due to the presence of negatively charged phenolic, carboxyl, and hydroxyl groups on the biochar surface [75], which can neutralize H^+^ in the soil solution, reduce the concentration of H^+^ in the soil solution, and thus increase soil pH. Field experiments showed that applying 22.5 t/hm^2^ of rice straw biochar to paddy field soil increased the soil pH by 0.30 units in the first year and by 0.26 units in the second year [76]. Moreover, since straw biochar contains a large amount of carbon, biochar can directly increase the soil organic matter content by carrying carbon itself, and it can also affect the formation of soil organic matter by changing the soil’s physical and chemical properties [77].

### 4.2. Soil Heavy Metal Activity

Compared to the removal of heavy metals from water bodies, there is less research on the impact of straw biochar on the availability of heavy metals in soil, which is related to the complexity of the soil environment. Studies have shown that the application of straw biochar can significantly reduce the bioavailability of heavy metals in soil. Straw biochar can adsorb metals in the soil, such as Cu, Cd, and Pb, thereby reducing the availability of these metals [78,79]. Most experiments indicate that the application of straw biochar leads to a decrease in the content of active heavy metals. The application of 10% maize straw biochar reduced the As, Cd, Cu, and Pb accumulation in the leaf (56.0%, 49.7%, 41.3%, and 59.7%), stem (50.0%, 39.2%, 36.1%, and 72.1%), and tuber (39.7%, 25.0%, 42.8% and 63.7%), respectively [80]. The type, concentration, and form of heavy metals can have varying impacts on soil remediation via biochar. For instance, Khan et al. demonstrated that biochar significantly lowers the bioavailable concentrations of lead and cadmium in the field. However, it has no impact on zinc, likely because of the distinct activities of different heavy metals [81]. In soils laden with multiple heavy metals, competitive adsorption among various heavy metals can lead to differential reactivity. For example, lead (Pb) may form complexes more readily on the surface of biochar compared to cadmium (Cd). Additionally, manganese oxides exhibit a notably stronger adsorption capacity for lead than for cadmium [82].

Zheng et al. found that the application of biochar significantly reduced the bioavailable contents of Cd, Zn, and Pb, while As increased [83]. Biochar promotes the reduction of As(V) to As(III), leading to an increase in the toxicity and mobility of As in contaminated fields [84]. Therefore, the different geochemical behaviors of cationic heavy metals such as Cd and As make it difficult for them to be co-immobilized.

## 5. The Immobilization Mechanism of Heavy Metals by Biochar

### 5.1. pH

With the increase in the application of straw biochar, the soil pH value can be significantly increased, which in turn leads to an increase in the hydrolysis of heavy metals, thereby enhancing the soil’s adsorption of heavy metals and accelerating the transformation of heavy metal forms in the soil towards oxidizable and residual states [85]. An increase in soil pH value may also increase the complexation of heavy metals, reducing the desorption of heavy metals from the soil, thus reducing the bioavailability of heavy metals [86].

### 5.2. Physical Adsorption

Biochar can adsorb heavy metals on its surface or diffuse into its pores, fixing heavy metals through interactions such as van der Waals forces [87]. Generally, a larger specific surface area and porosity facilitate the physical adsorption of heavy metals by biochar [88]. There are significant differences in the adsorption efficiency of biochar made from different straw types for heavy metals in soil. The adsorption efficiency of typical straw biochar for heavy metals in soil is shown in Table 3.

### 5.3. Electrostatic Adsorption

The surface charge of biochar facilitates the adsorption of heavy metals through electrostatic interactions, thereby immobilizing them [92]. The electrostatic adsorption of heavy metals by biochar is contingent upon the environmental pH and the biochar’s point of zero charge (pH_PZC_). When the medium’s pH exceeds the pH_PZC_, the biochar’s surface acquires a negative charge, enabling it to electrostatically adsorb cationic heavy metals. Conversely, when the medium’s pH is below the pH_PZC_, the biochar can electrostatically adsorb anionic heavy metals [93]. Additionally, an increase in pH can enhance the biochar’s adsorption of heavy metals, as higher pH values lead to a greater concentration of negatively charged sites on the biochar surface, which in turn strengthens the electrostatic attraction to cationic heavy metals.

### 5.4. Ion Exchange

The basic ions on the surface of biochar can be displaced by heavy metal ions, thereby fixing the heavy metals. At the same time, carboxyl groups and other oxygen-containing functional groups can also adsorb metal ions through ion exchange pathways [53]. The biochar’s highly electronegative surface grants it significant cation exchange capability and the capacity to emit cations like Ca^2+^ and Mg^2+^. These cations are subsequently able to engage in exchange reactions with heavy metal ions present on the biochar’s surface [94].

### 5.5. Chelation

The surface functional groups of biochar offer attachment points that enable the formation of complexes with heavy metals, effectively immobilizing them. This complexation process is notably pronounced in biochars that have a minimal mineral composition. Take, for instance, biochar produced from agricultural crop residues, which predominantly captures heavy metals through the creation of surface complexes [95]. Compared to hydroxyl groups, the thiol groups on the surface of high-sulfur biochar have a stronger complexation ability for Hg, Cd, Mn and others [96]. Thiol-modified rice straw biochar can be prepared using catalytic transesterification with thiol ethanol, which increases the adsorption capacity for Cd by three times (45.13 mg/g). Cd can form stable Cd-sulfur complexes that are not easily desorbed, whereas 57% of the Cd adsorbed by unmodified biochar can be desorbed [58].

### 5.6. Precipitation

The mineral components in the ash fraction of biochar can undergo precipitation reactions with heavy metals, thereby immobilizing heavy metals. In rice straw biochar, C_2_O_4_^2−^ and CO_3_^2−^ can form PbC_2_O_4_ and Pb_3_(CO_3_)_2_(OH)_2_ precipitates with Pb, which are the main mechanisms for fixing Pb [97]. Additionally, Pb can also form precipitates such as chlorides, phosphates, and sulfates with other minerals contained in biochar [98]. In fact, except for Pb, most metal elements can also combine with the mineral elements contained in biochar to form precipitates [99]. Xu et al. investigated the role of precipitation in the adsorption of cadmium (Cd) by biochar derived from cow manure. Their findings indicated that precipitation was responsible for 88% of the total adsorption process. Specifically, the contribution of soluble CO_3_^2−^ to this process was found to be more significant than that of PO_3_^4−^ [100].

### 5.7. Redox Reaction

Biochar is rich in various functional groups, such as quinones and phenolic hydroxyl groups, which enable it to store and transfer electrons. This capability allows biochar to oxidize As(III) or reduce Cr(VI), thereby effectively immobilizing heavy metals. For instance, the quinone moieties in biochar act as electron acceptors, while phenolic and hydroquinone moieties serve as electron donors, facilitating electron transfer and redox reactions that contribute to the immobilization of heavy metals [101,102]. Hydrogen and oxygen atoms in the molecular structures of quinone and phenolic hydroxyl groups on the surface of the aromatic ring of biochar undergo electron delocalization, leading to the presence of unpaired electrons in atomic orbitals, thus forming persistent free radicals (PFRs) [103]. Mohan et al. discovered that the phenolic hydroxyl groups present in biochar can serve as electron donors for Cr(VI). These groups are subsequently oxidized to quinone groups, which then engage in complexation reactions with the adsorbed Cr(VI) [104].

## 6. The Application of Biochar in Field Remediation and Its Potential Risks

### 6.1. Field Remediation Application

#### 6.1.1. Application Rate

Biochar is mainly applied in two ways: one is a one-time large dose return to the field, and the other is in batches. A one-time large dose return to the field may introduce too many pollutants into the soil, exceeding the soil’s own environmental capacity and having a negative impact on soil microorganisms [105]. When applying in batches, reference can be made to the GB4284-2018 “Agricultural Sludge Pollutant Control Standard” [106]. When the sludge meets the standard, the annual application should not exceed 30.0 t/hm^2^, and continuous application should not exceed 20 years [107]. From the current state of research, it appears that biochar application rates in field trials typically range from 1.5 to 40 t/hm^2^. Generally speaking, as the amount of biochar applied increases, the reduction in bioavailable heavy metals in soil and crops increases, and crop yield increases. Houben et al. used different biochar application rates (1%, 5%, 10%) to remediate Cd, Zn, and Pb-contaminated soil in Belgium. After 56 days of cultivation, the CaCl_2_ extractable heavy metals in the soil decreased with the increase in biochar application [108]. However, Bian et al. reported a 1.81% reduction in wheat yield after using a higher amount of biochar (40 t/hm^2^) to remediate contaminated soil [109]. This is possibly due to the strong adsorption capacity of biochar, which reduces the availability of nutrients in the soil. It is also possible that the addition of biochar increases soil pH, which leads to the volatilization of NH_3_ [110]. In addition, studies have shown that biochar can inhibit nitrification by adsorbing large amounts of chemicals such as phenol and terpenes, thereby reducing the concentration of alkaline nitrogen in soil [111]. Of course, this change is closely related to the properties of the soil, and the impact varies greatly among different types of soil.

#### 6.1.2. Application Method

Biochar is generally applied in engineering by spreading on the surface or mechanically spot-applying it, turning it into the soil layer of 15 to 30 cm. Studies have shown that increasing the depth of application can significantly improve crop yields, but it can also increase the accumulation of heavy metals in crops [112]. This may be due to the fact that deep plowing improves the pH and nutrient conditions of crop rhizosphere environment through biochar, promoting crop growth. However, deep plowing may cause heavy metals to move downwards and dilute biochar, weakening the fixation of heavy metals and increasing crop absorption of heavy metals.

### 6.2. Potential Risks

Biochar can improve soil aeration and water-holding capacity, decrease soil nutrient and the toxicity of soil pollutants, and thereby increase crop yields [106]. However, these benefits primarily come from fresh biochar, and the quantity and quality of biochar can change due to continuous aging [113,114]. Relevant studies have reported that, after 5 years of biochar application, the mass loss rate of biochar in the 0–20 cm soil layer is close to 40% [115]. Aging affects the adsorption of pollutants by biochar. Guo et al. found that, after aging paddy husk biochar in the dark and constant humidity for 300 days, the maximum adsorption capacity of the aged biochar for Cu decreased by 3.06% [116]. Moreover, during the long-term aging process of biochar, heavy metals adsorbed by biochar can release again [100]. In the field applications, due to differences in soil environment and climate, the impact of aged biochar will be difficult to determine, potentially posing certain environmental risks [117,118]. It is particularly important to pay attention to the fact that straw biochar, in terms of contamination remediation, may lead to an increase in pollutant activity due to aging, which requires enhanced monitoring.

Biochar particles, especially nanoscale biochar particles, may carry pollutants and migrate along the soil profile, posing a potential risk to groundwater [119]. Wang et al. studied the migration of biochar in saturated granular media using glass chromatography columns and found that the mobility of biochar is affected by particle size and preparation temperature, with mobility decreasing as particle size increases, and biochar prepared at higher temperatures exhibiting lower mobility [120]. Since the porous medium quartz sand used in the experiment does not represent the actual soil system, and factors such as soil ion strength, pH value, natural organic matter, biochar application method, burial depth, and dosage will all affect the migration of biochar, further research is needed on the mechanisms of biochar migration and retention in different types of soils under natural conditions.

## 7. Application Potential Analysis

China has abundant crop straw resources. According to the Ministry of Agriculture’s 2010 “National Crop Straw Resource Survey and Evaluation Report”, the theoretical resource volume of crops in China in 2009 was 820 million tons, with 215 million tons of unused resources. The unused straw is mainly abandoned and burned on-site [121]. According to the biochar application rate recommended by the IBI of 5–50 t/hm^2^, it would require 135 to 1350 million tons of biochar resources to remediate 1.8 million hm^2^ of contaminated arable land. Calculating with a biochar yield of 30–40%, this would require 450 to 4500 million tons of biomass. This amount is 2 to 25 times the volume of China’s unused biomass resources. On one hand, straw carbonization can reduce air contamination caused by straw burning, and, on the other hand, it can achieve the efficient utilization of solid waste. Therefore, utilizing crop straw for biochar production is a development direction worth paying attention to.

In the process of engineering remediation, the cost of stabilizing agents is a significant limiting factor. Reports indicate that the production cost of biochar is USD 113/t [122]. Ahmed et al. statistically concluded that the production cost of biochar(including wages, storage, production, etc.) is between USD 98–353/t [123]. Considering the average costs of other stabilizing agents at USD 158/t (lime), USD 25.4/t (coal fly ash), USD 116/t (FeS), and USD 142/t (Na_2_S), biochar has a certain price advantage, and the choice of biochar as a soil contamination stabilizing agent has a certain prospect.

## 8. Conclusions and Future Prospect

Biochar has characteristics such as a high pH value, large specific surface area, and abundant surface functional groups, which give it a strong ability to immobilize heavy metals. However, during the in situ remediation process, the pH of biochar and soil will gradually decrease, leading to a risk of re-release of fixed heavy metals. The properties of biochar can be enhanced through the use of acids and bases, oxidizing agents, organic solvents, and metal salts or oxides, with the type or concentration of the modifying agent affecting the modification effect. Biochar directly immobilizes heavy metals through physical adsorption, electrostatic adsorption, complexation precipitation, and redox reactions. The main adsorption and immobilization mechanisms vary for different heavy metals. Considering the complexity of biochar properties and soil media, a variety of factors such as the properties of biochar, application rate, soil contamination conditions, and potential interactions between biochar, heavy metals, and soil should be comprehensively considered before soil remediation.

It is necessary to research the manufacturing equipment for straw biochar to further reduce costs and minimize the production of pollutants during the preparation process, especially the rational recovery and utilization of by-products such as tar. It is also necessary to research the combined effects of straw biochar with other amendments or fertilizers. Since biochar is powdery and not conducive to direct broadcasting, its use in combination with other amendments or fertilizers can facilitate application and compensate for the shortcomings of biochar’s broadcasting method and efficacy. It is necessary to conduct follow-up studies on biochar applied to the soil, establish long-term positioning experiments, and determine the long-term impact of straw biochar on the agricultural soil environment.

## Figures and Tables

**Table 1:** Properties of biochar from different sources.

Feedstock Type	Temperature	Ash Content	pH	Elemental Composition				References
				C	H	N	O	
Rice straw	300	25.8	9.50	55.7	3.26	1.40	39.6	[42]
	500	30.3	12.3	64.3	2.12	0.86	32.7	
	700	33.7	12.8	68.7	1.33	0.35	29.5	
Maize straw	300	16.3	9.84					[43]
	450	22.3	10.5					
	600	27.2	11.4					
Wheat straw	500	24.6	10.3	66.4	3.36	1.19	4.43	[44]
Hardwood	600	2.98	6.96	78.9	3.61	0.67	13.8	
Softwood	550	1.25	7.91	85.5	2.77	0.10	10.4	
Sawdust	700	3.86	8.90	47.3	0.63	0.85	2.06	[45]

**Table 2:** Preparation and application of modified biochar.

	Modified Methods	Feedstock Type	Application	Specific Surface Area m^2^/g	Mechanism	References
Physical modification	NH_3_/CO_2_ modification	Cotton stalks	CO_2_ removal		Increase the specific surface area and the content of nitrogen-containing compounds to enhance the physical/chemical adsorption capacity	[48]
Chemical modification	HCl/HNO_3_/NaOH	Rice husk	Tetracycline removal	34.4–117.8	Increase specific surface area, -oxygen-containing functional groups and π-π bond interactions	[54]
	Ca and Mg loaded biochar modification	Maize cob	Recovery of phosphorus from biogas fermentation liquid	126.5–487.5	Increase in functional groups and nanoscale MgO particles	[55]
	Methanol modification	Rice husk	Tetracycline removal	51.9–66.0	Increase in oxygen-containing functional groups and influence on π-π electron transfer	[56]
Organic modification	Sodium alginate	Rice straw	Pb removal	42.1–110.8	Increase the surface area and porosity	[57]
	β-Mercaptoethanol /acetic anhydride/ concentrated sulfuric acid	Rice straw	Pb and Cd removal	0.34–7.82	Introducing hydroxyl, mercapto, and other groups	[58]
	Citric acid	bean straw	Pb and Cd removal		Increase the number of oxygen-containing functional groups	[59]

**Table 3:** The adsorption efficiency of typical straw biochar for soil heavy metals.

Straw Type	Heavy Metal		Preparation Condition		Dosage/g/kg	Adsorption Efficiency/%	References
	Heavy Metal Type	Heavy Metal Concentration mg/kg	Temperature/ °C	Time/h			
Rice straw	Cd	50	500	2	50	57.1	[89]
	Pb	100				74.6	
Wheat straw	Cd	58.3	900	2	30	50	[90]
	Pb	3074				94.8	
	Zn	4234				22.3	
Corn straw	Hg	4.0	400	10	30	9.88	[91]
	Cd	3.0				10.1	
	Pb	800				11.0	

## Data Availability

The data used to support the findings of this study are available from the corresponding author upon request.
